# Diversified legume-oilseed cropping system for synergistic enhancement of yield and water use efficiency in rainfed areas of semi-arid tropics

**DOI:** 10.1371/journal.pone.0317373

**Published:** 2025-02-12

**Authors:** V. Visha Kumari, Gopinath K. A., Sarath Chandran M. A., A. K. Shankar, Suvana S., Manoranjan Kumar, B. M. K. Raju, N. Jyothilakshmi, Savitha Santosh, G. Venkatesh, K. Sriram, B. Sunitha, Prasanna G. K, Subrata Bag, M. S. Rao, V. K. Singh

**Affiliations:** ICAR- Central Research Institute for Dryland Agriculture, Hyderabad Telangana, India; ICAR - IIFSR: ICAR - Indian Institute of Farming Systems Research, INDIA

## Abstract

This study explores the development of diversified legume-oilseed cropping systems aimed at enhancing yield and water-use efficiency in rainfed areas of semi-arid tropics. Dryland agriculture, often limited by mono-cropping practices and erratic rainfall, necessitates innovative approaches for crop intensification and sustainability for the future. The integration of legumes and oilseeds into double cropping systems offers a viable solution for optimizing land use and improving productivity under precipitation-limited conditions. The research was conducted at the Gungal Research Farm of ICAR-Central Research Institute for Dryland Agriculture during the 2022-2024 cropping seasons. Six cropping systems, with and without rainwater management, were evaluated. Key findings indicate that rainwater management especially during the flowering and pod filling stage significantly enhanced crop growth, biomass accumulation, and overall yield, with safflower and sesame showing the highest adaptability to moisture stress. In terms of green gram equivalent yield, cowpea-sesame system with rainwater management achieved the highest yields, recording 1655 kg ha^-1^ in 2022 and 1362 kg ha^-1^ in 2023, highlighting the critical role of rainwater management in enhancing crop productivity in semi-arid regions. The study identified a diversified legume-oilseed cropping system as a means to achieve sustainable agricultural production in semi-arid regions.

## 1. Introduction

Dryland agriculture, often limited by mono-cropping practices and erratic rainfall, necessitates innovative approaches for crop intensification and sustainability for the future. This practice of mono-cropping results in lower cropping intensity. The reliance on long-duration crop varieties during the monsoon season and change in the climate exacerbates this issue, leading to suboptimal land use. With the ever-increasing demand for agricultural produce and the decreasing per capita availability of agricultural land, there is a pressing need for both temporal and spatial intensification of crops

Intensification of crops production in drylands can be achieved through intensification of crop through diversification. As much as 5–10 per cent of the potential cropping intensity may be realized over the next 35 years in less-developed countries if minimum requirements for various inputs are met [1]. Diverse cropping systems enable this intensification by cultivating two or more crops in the same field contemporaneously on time after another [2]. This underscores the importance of efficient cropping systems specific to region, influenced by climate, soil, and socio-economic factor.

Changing climate and its impact is well observed in various crops [3,4] and agricultural systems [5]. Hence, the farmer’s selection of an appropriate cropping system and crop cultivar, especially in rainfed regions could be one strategy for adaptation to changing climatic conditions [6,7]. Double Cropping, that is raising two crops in one year instead of just one increases the productivity per unit area and can help to provide more stable annual income based on rainfed conditions where water availability is limited provided we select appropriate crops. Diversified crops in the system also reduce the negative environmental impacts and loss of biodiversity too [8,9].

This approach was feasible when early rains allowed the establishment of the first crop and lasted long enough for the second crop to mature [10]. However, in the recent years with the change in climate, late onsets, poor distribution and early withdrawal of monsoon has become a common phenomenon. While double-crop farming presents greater production risks due to narrower weather tolerances, it offers significant benefits in terms of crop diversification, improved nutrient cycling, and enhanced water-use efficiency [11] the success of raising successful double crop in rainfed regions only becomes possible if proper management of received rainwater is made. Double cropping system has been suggested and intensively investigated over the last years [12,13,14]. Replacing long-duration crops with short-duration, high-yielding varieties can further optimize this system. There were many works already done on double cropping. All India coordinated research on Dryland Agriculture and ICRISAT are pioneers in identifying new systems for sustainability. To quote a few, the work by [15] reports demonstrated that cropping during the rainy season is technically feasible in vertisols, and that grain productivity of double cropped sorghum +  chickpea (SCP–SCP) and mung bean +  sorghum (MS–MS) sequential systems were higher than their conventional counterparts with rainy season fallow. Similarly, [16] has given a brief of different double cropping systems available in India. However, all these works focus on cereal based system and shows its suitability in vertisols.

The integration of legumes and oilseeds into a system is particularly advantageous for dryland regions which characterized by limited water availability and erratic rainfall patterns [17]. Legumes, such as cowpea, black gram, and green gram, contribute to soil fertility through atmospheric nitrogen fixation, reducing the need for synthetic fertilizers and enriching soil organic matter [18,19]. Legumes also provide essential proteins, contributing to food security and nutrition [20]. Oilseeds like safflower and sesame enhance crop rotations and farm income, producing high-value oils for consumption and industrial applications. Legumes -oilseed system is advantages for their importance to improve the soil health along with meeting the food and nutrient requirement. However, successful implementation of double cropping systems in drylands necessitates a comprehensive understanding of agronomic practices, cropping calendars, and regional environmental conditions [21,22]. Scientific research plays a crucial role in optimizing these systems, addressing challenges such as crop selection, pest and disease management, and water conservation strategies. Effective rainwater management, particularly the harvesting of water during the rainy season for use in the *rabi* season (post monsoon season), is critical for the success of the second crop. Hence an attempt was made to identify a climate-adaptive diversified legume-oilseed cropping systems for rainfed areas of semi-arid regions to provide high annual crop yields for effective crop production through persistent soil protection and reduced nutrient losses.

## 2. Materials and methods

### 2.1. Site description

The study was carried out at the Gungal Research Farm of ICAR- Central Research Institute for Dryland Agriculture (17^o^ 05’ N, 78^o^ 39’E) between 2022-2023 and 2023-2024 with 6 legume - oilseed cropping systems with and without rainwater management.

### 2.2. Treatment details

The crops (both legumes and oilseed) used as treatments are presented in Table 1. The legumes were sown during the *kharif* and the oilseed crops were sown after the harvest of the legume crop (October). The crops under rainwater management were given 2 supplementary irrigations (5 mm) from the water harvested during *kharif* season in the year 2022-2023 and 2 supplementary irrigation in the year 2023-2024. The soil in the experimental field had a sandy loamy texture, with a pH of 6.04, and EC 0.12 ds m^-1^, the soil had medium fertility characterized by available nitrogen (215.48 kg ha^-1^), available phosphorus (26.73 kg ha^-1^), available potassium (218.34 kg ha^-1^), organic carbon (0.45%). The experiment was laid out in a Randomized Block Design and replicated thrice. The fertilizers were applied as per the recommendation. The crop, variety, spacing, fertilizer, sowing and harvesting time of the crops are given in Table 2. Figure 2a and 2b shows the amount of rainfall recorded during the two growing seasons for the cropping systems.

**Table 1:** Treatments/systems.

Monsoon Crop	Post monsoon crop	
	With rainwater management	Without rainwater management
Green gram (GG)	Safflower (SF+)	Safflower (SF-)
Cowpea (CP)	Safflower (SF+)	Safflower (SF-)
Black gram (BG)	Safflower (SF+)	Safflower (SF-)
Green gram (GG)	Sesame (SS+)	Sesame (SS-)
Cowpea (CP)	Sesame (SS+)	Sesame (SS-)
Black gram (BG)	Sesame (SS+)	Sesame (SS-)

**Table 2:** Management practices of crops under study.

Crop	Variety	Seed rate (kg ha^-1^)	Spacing (cm)	Fertilizer (kg ha^-1^)	Sowing	Harvesting
Green gram	WGG37	10-15	30 × 10	20:50:0	July	October
Cowpea	TPTC29	20-25	40 × 20	20:40:0	July	October
Black gram	JS9305	20-25	30 × 10	20:50:0	July	October
Safflower	ISF-64	12-15	45 × 15	40:25:20	October	February
Sesame	Shweta *til*	5-7	45 × 15	40:20:20	October	February

**Fig 1:**
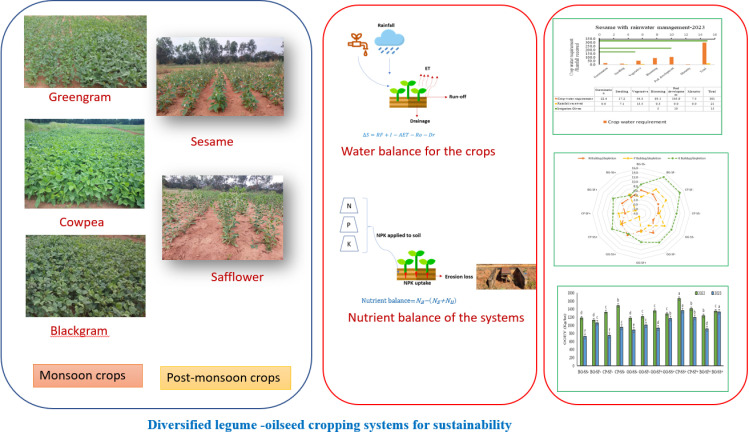
Diversified legume-oilseed cropping system.

**Fig 2:**
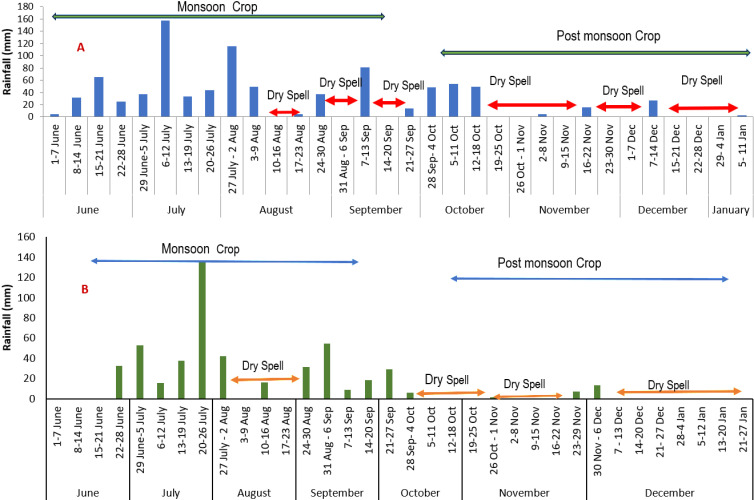
Rainfall (mm) recorded during the two growing seasons (A 2022-23 and B: 2023-24).

### 2.3. Growth parameters

#### 2.3.1. Leaf Area Index (LAI).

The leaf area was measured using LICOR (LI-3100C). The leaf area Index was calculated by using the following formulae given by [23].


Leaf area=(Leaf area)/Ground area(spacing)

#### 2.3.2. Biomass accumulation (g/plant).

Various stage wise biomass was recorded in the different growth stages of crops Samples were dried at 65 ºC to attain constant weight and average dry weight was calculated and expressed in grams per plant.

### 2.4. Physiological parameters

#### 2.4.1. Chlorophyll and carotenoids content.

Leaf chlorophyll and carotenoids were estimated using a UV visible Spectrophotometer (Motras Scientific, India) using the methodology given by [24]. The results are expressed in mg g^-1^ of fresh leaf weight.

#### 2.4.2. Proline.

Free proline contents in the leaves of the post monsoon crop (*rabi* crop) was determined in the flowering stage using the method of [25].

#### 2.4.3. Relative leaf water content (RLWC).

Collected leaves were cut into pieces and recorded fresh weight (FW), turgid weight (TW) and dry weight (DW) according to the methodology given by [26]. The RLWC was expressed as:


RLWC=  (FW-DW)/(TW-DW)  ×100

### 2.5. Nutrient balance

Nutrients (NPK) balance of different systems was calculated based on the method described by [27]. The nutrients (NPK) added (N_a_), uptake by the plants (N_u_), lost from soil through erosion (N_s_) were taken into consideration for the calculation.


$${\text{Nutrient balance=N}}_{\text{a}}\text{-}\left({\text{N}}_{\text{s}}{\text{+N}}_{\text{u}}\right)$$

### 2.6. Apparent nutrient balance sheet

At the end of the two-year experiment, the changes in nutrient balance were determined by subtracting the nutrients extracted by the crops from those added as fertilizer [28].

### 2.7. Water budgeting

Robinson and Hubbard water balance method [29] was used to calculate the crop phenology-based water requirement as well as water availability for each crop grown. Potential evapotranspiration (*ET*_P_) was calculated using Hargreev’s method. Potential transpiration was estimated by employing crop coefficient (*K*_c_) derived from the literature [30,31,32].

### 2.8. Water use efficiency

For each system rain water use efficiency (WUE) was calculated as given below


WUE=Yield/Moisture available (rainfall + supplementary irrigation)

WUE is expressed in (kg ha^-1^-mm), yield kg ha^-1^ and moisture available in mm

WUE measures the yield produced by a system for each mm of rainfall received for the monsoon crop. However, for the post monsoon crop it would take consideration of the supplementary irrigation provided.

### 2.9. Crop yield

The whole plot was harvested to estimate the yield of the crop. The overall productivity of each crop sequence was assessed by calculating their economic green gram equivalent yield (GEY) using the formula given below.

GEY = Yield of each crop (kg ha^-1^) x Economic value (Rs. kg^-1^)/price of green gram (Rs. kg^-1^)

**Table:** The price used for calculating GEY is given below

Crop	Price in 2022 (Rs. kg ^ -1 ^)	Price in 2023 (Rs. kg ^ -1 ^)
Blackgram	66	69
Greengram	78	75
Cowpea	59	62
Safflower	53	59
Sesame	78	96

* 1 Rupee= 0.12USD.

### 2.10. Statistics

The soil analysis results were presented as means with standard deviations from three replicates (n). For statistical analysis, IRRI Stat (2.0.1) and ANOVA [33] were used. Treatment means were compared considering the significant differences using Tukey’s HSD post hoc comparisons (p ≤ 0.05).

## 3. Results and discussion

### 3.1. Growth parameters

**Fig 3:**
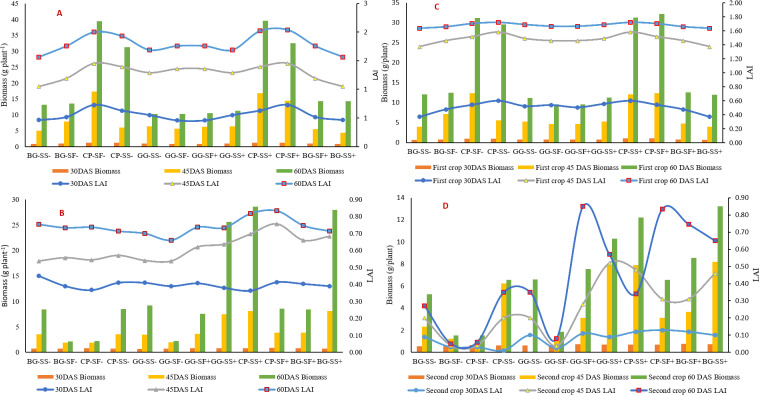
Biomass and LAI of various cropping systems (monsoon and post monsoon crops) in the year 2022 and 2023 (A: Monsoon crop 2022, B: Post monsoon crop 2022, C: Monsoon crop 2023, D: Post monsoon crop 2023).

In both the years 2022 and 2023, there was a significant increase in Leaf Area Index (LAI) and biomass from 30 to 60 days after sowing (DAS) showing a linear growth of various crops sown. In the year 2022, cowpea showed the highest values for both LAI (0.83, 1.55) and biomass (1.25 g plant^-1^, 17.29 g plant^-1^) at 30 and 45 DAS (Fig 3a) respectively. The highest value highlights rapid canopy development and substantial biomass accumulation in cowpea. This early vigour is critical for effective light interception, which has been well-documented as a key factor in enhancing photosynthetic efficiency and biomass production [34,35]. However, the highest LAI at 60 DAS was observed in black gram and green gram (1.86), while cowpea had the highest biomass (39.46 g plant^-1^) suggesting a more efficient conversion of intercepted light into biomass. This finding aligns with previous studies indicating that different crops have varying growth strategies and resource allocation patterns [36,37]. Among the post-monsoon crops in 2022, sesame without rainwater management showed the highest LAI at 30 DAS (0.45), while safflower exhibited the highest biomass (0.89 g plant^-1^). At 45 DAS, safflower with rainwater management recorded the highest LAI (0.76) (Fig 3b), indicating the positive impact of rainwater management on crop growth [38]. By 60 DAS, the highest LAI was again recorded in safflower with rainwater management (0.83), while sesame with rainwater management achieved the highest biomass (28.56 g plant^-1^), demonstrating the critical role of water management in optimizing crop productivity [39].

In the year 2023, a linear increase in Leaf Area Index (LAI) and biomass was observed in all crops. Cowpea recorded the highest LAI and biomass at 30, 45, and 60 DAS (0.60, 1.58, 1.72 and 1.05 g plant^-1^,12.35 g plant^-1^, 32.21 g plant^-1^) (Fig 3c). The year 2023, was extremely dry and the water available for the crops was one tenth of the water requirement (Fig 2b). In the year 2023, safflower showed the highest LAI at 30 (0.13), whereas at 45 DAS sesame showed the higher LAI (0.52) (Fig 3d). The supplemental irrigation provided during critical growth stages of safflower (flowering and seed development) significantly enhanced LAI, underscoring the importance of water management in mitigating stress and promoting growth [40]. At 45 and 60 DAS, sesame with rainwater management recorded the highest biomass (8.21 g plant^-1^ and 13.21 g plant^-1^, respectively (Fig 3d), reaffirming the benefits of effective water management practices [41]. The crop raised with no rain water management was completely lost in the year 2023 due to the continuous dry spells (Fig 1b). The Fig 2 shows the continuous dry spell the crop has experienced in the year.

### 3.2. Physiological parameters

**Fig 4:**
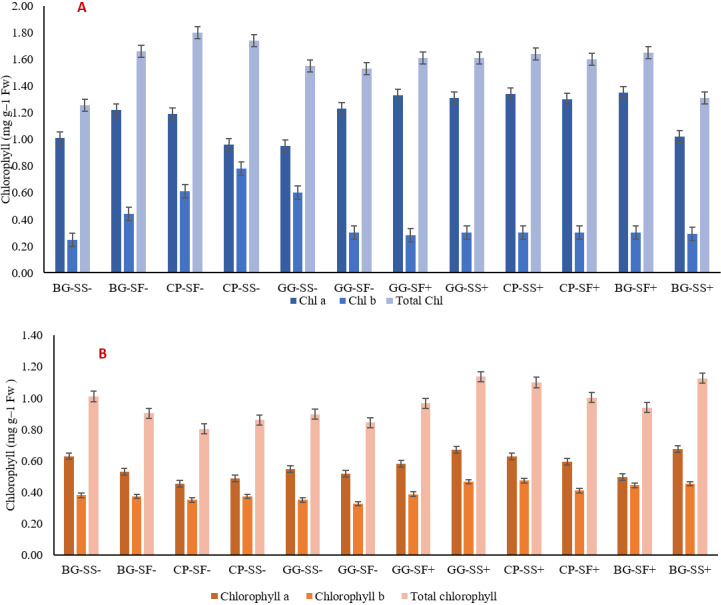
Chlorophyll a, b and total chlorophyll content of different cropping systems in the year 2022 (A) and 2023 (B).

The physiological parameters are presented here only for the post monsoon crops as there was no significant difference among the crops raised during the monsoon. Post-monsoon crops provided crucial insights into the adaptability and resilience of different cropping systems under varying environmental conditions. Safflower exhibited the highest total chlorophyll content, chlorophyll a and chlorophyll b measuring 1.80 mg g^-1^ fresh weight (FW), 1.35 mg g^-1^ FW and 0.78 mg g^-1^ FW in 2022, whereas sesame recorded highest total chlorophyll, chlorophyll a and chlorophyll b in 2023 (1.33 mg g^-1^ FW) (Fig 4a and 4b). Conversely, the lowest chlorophyll content was found in the sesame in black gram-sesame system without rainwater management in 2022 (1.26 mg g^-1^ FW) and safflower in cowpea-safflower system without rainwater management in 2023 (0.80 mg g^-1^ FW, Fig 4a and 4b). The results showed that hardiness of safflower to stress and better rainwater management to provide supplementary irrigation during critical stages can maintain higher photosynthetic efficiency and result in an efficient cropping system.

**Fig 5:**
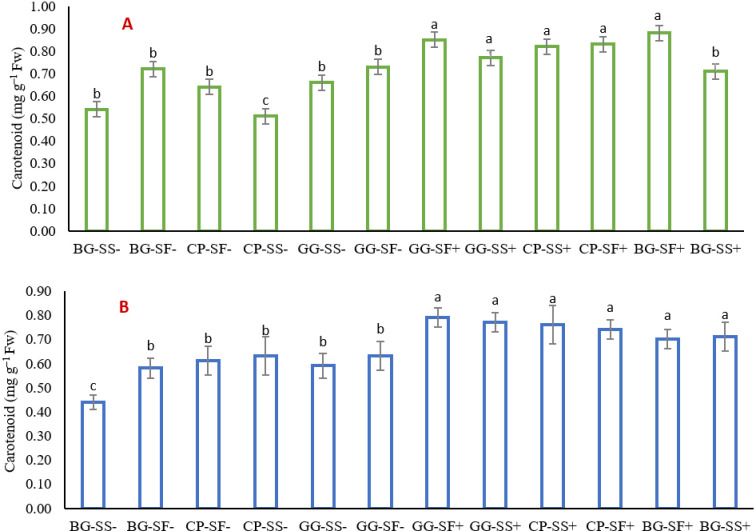
Carotenoid content of different cropping systems in the year 2022 (A) and 2023(B).

In contrast to chlorophyll content, safflower crop in blackgram-safflower with rainwater management and greengram-safflower with rainwater management showed the highest carotenoid content in both the years 2022 (0.88 mg g^-1^ FW in 2022) and 2023 (0.79 mg g^-1^ FW), respectively showing its adaptation to dry spells (Fig 5a and 5b). Lowest carotenoid content was observed in sesame in cowpea-sesame system without rainwater management and black gram-sesame system without rainwater management respectively in 2022 (0.51 mg g^-1^ FW) and 2023 (0.44 mg g^-1^ FW). Carotenoids play a crucial role in protecting chlorophyll from photooxidative damage and are also involved in the photosynthetic process [42].

**Fig 6:**
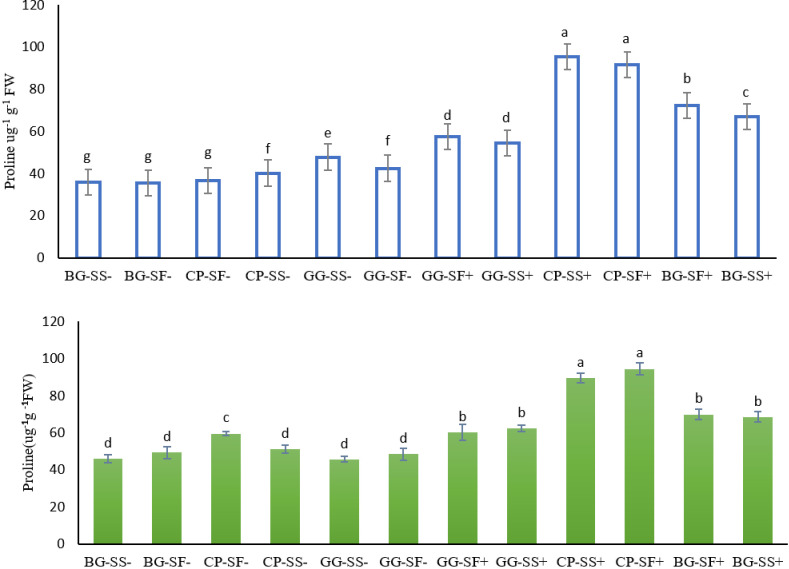
Proline content of different cropping systems in the year 2022 (A) and 2023(B).

**Fig 7:**
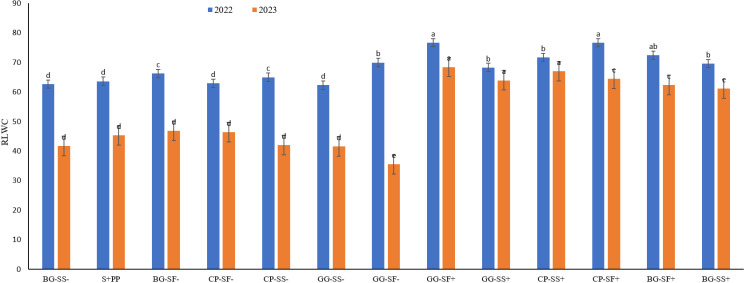
Relative water content of cropping systems in 2022 and 2023.

Proline plays a crucial role in osmoregulation and in the accumulation of low molecular weight metabolites, including sugars, organic acids, and amino acids [43,44,45]. Proline is key osmoprotectant [43] that helps in ROS scavenging, safeguards cell membranes from oxidative damage. The data from this study revealed that proline content in the cropping systems ranged from 35.5 mg g^-1^ FW to 95.5 mg g^-1^ FW across both years (Fig 6a and 6b). The highest proline content was found in the sesame in cowpea-sesame with rainwater management crop under stress conditions in 2022 (95.5 mg g^-1^ FW), while safflower in cowpea-safflower with rainwater management recorded in 2023 (94.5 mg g^-1^ FW). Similarly, the lowest proline content was observed in safflower in black gram-safflower without rainwater management in 2022 (35.5 mg g^-1^ FW), while sesame recorded in the green gram-sesame without rainwater management in 2023 (45.6 mg g^-1^ FW). Safflower being a hardy and drought tolerant crop showed increased amount to proline and carotenoid production. Safflower raised with rainwater management were also better in maintaining better osmoregulation when compared to the crops raised without rainwater management (supplementary irrigation). These observations were also correlated with the RLWC of the particular stage. Highest relative water content recorded in the safflower crop in greengram-safflower with rainwater management (76.7% and 68.4%) both the years. While the lowest relative water content was with sesame in greengram-sesame without rainwater management in 2022 (62.3%) and safflower in greengram-safflower without rainwater management in 2023 (35.5%, Fig 7). Safflower being a hardy crop was able to retain its leaf water under stress conditions resulting in higher relative water content whereas sesame was unable to maintain its water content resulted into lower RLWC [45].

### 3.3. Nutrient budgeting

The apparent nutrient balance is a crucial indicator for evaluating the sustainability of cropping system practices [46]. In the experiment, nutrient applications to various cropping sequences ranged from 60-120 kg N ha^-1^, 40-75 kg P ha^-1^, and 40-75 kg K ha^-1^. The initial available nitrogen before the double cropping systems was 215 kg N ha^-1^. After two years of experimentation, the available nitrogen was 203-214 kg ha^-1^, available phosphorus was 14.1-22.1 kg ha^-1^, and available potassium was 205.5-210.2 kg ha^-1^ after crop uptake and soil losses.

#### 3.3.1. Nitrogen dynamics.

**Fig 8:**
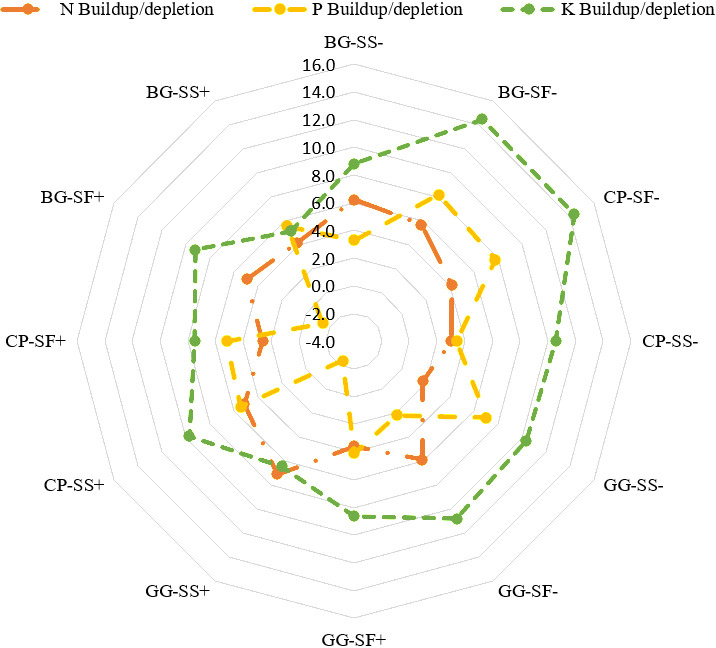
Nutrient budgeting of different systems after two years of study period.

Identifying systems with nutrient build up or deficiencies allows to understand and develop strategies to improve soil fertility and enhance crop productivity. After two years of experiment, the highest nitrogen build-up was observed in the greengram-sesame system with rainwater management (7.1 kg ha^-1^, Fig 8). The apparent nitrogen balance indicated a negative balance for all the systems. The systems that exhibiting negative balances, indicates a higher nitrogen loss or uptake by the crops [47]. According to the literature, legumes may obtain 54-70% of their nitrogen needs through biological nitrogen fixation (BNF) [48]. Therefore, considering the potential contribution from BNF, the reported negative nitrogen balance might be overestimated and may not accurately represent the depletion of soil nitrogen reserves.

#### 3.3.2. Phosphorus dynamics.

The initial phosphorus level was 26.73 kg ha^-1^, and after two years, the highest phosphorus build-up was observed in the black gram-safflower system without rainwater management (8.2 kg ha^-1^, Fig 8). The apparent balance sheet, calculated by subtracting nutrient removal by crops from nutrient additions from various sources, showed positive phosphorus (P) balances. The highest apparent phosphorus balance was found in the black gram-safflower system with rainwater management, with a balance of 59.1 kg ha^-1^. Phosphorus is a major nutrient for legumes, as it fixes atmospheric nitrogen and lower their dependence on nitrogen and potassium., the availability of P to the crops depends on root growth and its interaction with the abiotic and biotic components of soil [49]. These results indicate that phosphorus management is critical in these legumes-oilseed based systems to avoid depletion and maintain soil fertility [50].

#### 3.3.3. Potassium dynamics.

Initial potassium level was 218.34 kg ha^-1^, and actual levels ranged from 191.9-203.0 kg ha^-1^. The potassium build-up increased in the cropping systems due to lower crop uptake, with the highest build up observed in the blackgram-safflower without rainwater management (14.5 kg ha^-1^, Fig 8) after the completion of two years. The apparent potassium balance was positive, indicating that crop potassium uptake was less than the amount added. The highest apparent potassium balance among the cropping systems was recorded in the greengram-safflower system with rainwater management (53.4 kg ha^-1^). This positive balance highlights the need for balanced fertilization and suggests that fertilizer recommendations should be based on crop demands for a specific yield target and the soil’s native nutrient supply capacity [51].

### 3.4. Water budgeting through water balance

**Fig 9:**
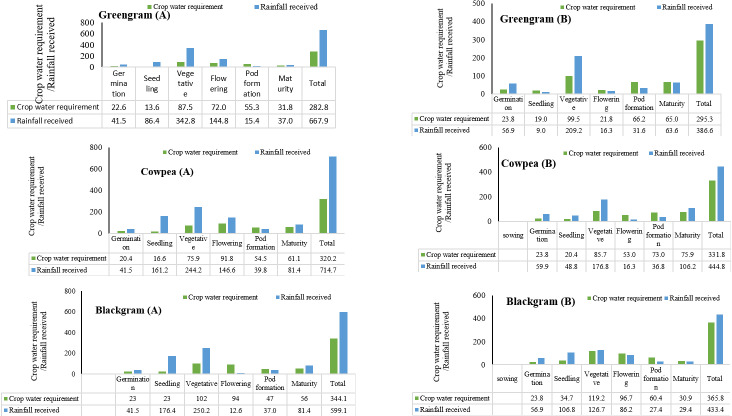
Robinsons-hubbard water balance for monsoon crops 2022 (A) and 2023 (B).

**Fig 10:**
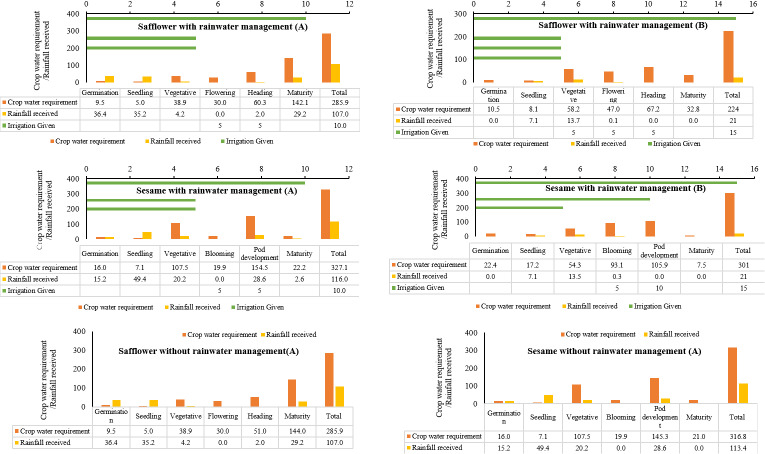
“Robinson-Hubbard Water Balance” for post monsoon crops in 2022(A) and 2023(B)3.5 Water use efficiency.

The Phenology based “Robinson-Hubbard Water Balance” method was used for accurate estimation of soil moisture availability and moisture requirement in each crop growth stage in both the years. As each stage of different crop has different water requirement, this estimation can actually help crops receive adequate water during critical growth periods if water is available. For each crop the water balance was calculated separately in both the years (Fig 9 to Fig 10). The total moisture/ water received by the crop may be adequate or more than the actual requirement of the crop. However, there may be deficient in moisture availability in specific stage that many hampers the yield of the crop. The analysis of rainfall distribution and its impact on various crops helped us to understand the importance of timely water availability during critical growth stages. The findings demonstrate that while overall water availability is important, the distribution of rainfall across different growth stages is crucial for optimal crop yield. Effective rainwater management and supplementary irrigation can help us to some extend to manage the vagaries of climate change and its effect on crop production.

In our study, we found such very important results. In 2022, the greengram crop received a total of 667.9 mm of rainfall, significantly exceeding its actual water requirement of 282.8 mm (Fig 9a). However, the crop experienced water stress during the critical pod formation stage due to insufficient rainfall (15.4 mm), leading to a noticeable impact on yield. This aligns with previous studies indicating that water stress during reproductive stages can severely affect yield [52]. The trend remained the same with blackgram (Fig 8a and 8b). Similarly, cowpea crop in 2022 received 714.7 mm of rainfall, which was well above its water requirement of 320.2 mm. Despite this, water stress occurred during the pod formation stage (39.8 mm), leading to fewer pods per plant (Fig 9a and 9b). In 2023, the crop experienced water stress during flowering (16.3 mm) and pod formation (36.8 mm) stages, despite receiving a total of 444.8 mm of rainfall, meeting the overall water requirement of 331.8 mm. These findings also reassure the importance of critical stage water requirement emphasizing that water stress during flowering and pod formation stages critically affects yield [53,54,55]. This can be used in the future to decide the critical stage of irrigation requirement for various crops.

The post monsoon crop, i.e., sesame and safflower were drastically affected by the dry spells. In 2022, the safflower crop received 107 mm of rainfall, significantly below its requirement of 285.9 mm (Fig 10a and 10b). Supplementary irrigations during the flowering and heading stages helped to achieve some yield. In 2023, the crop received only 21 mm of rainfall against a requirement of 224 mm, necessitating three supplementary irrigations. Despite these efforts, the crop’s water needs were not fully met, resulting in an early completion of the life cycle and reduced yield. Though we provided three supplementary irrigation, the crop was not able to withstand the stress and resulted in low yield. It is a known fact that supplementary irrigation can mitigate some yield loss but may not fully compensate for severe water deficits [56]. In crop production, rather than aiming for the maximum yield per unit area through full irrigation, it may be more effective to limit the number of irrigations or the amount of irrigation water. This approach allows for minor yield reductions per unit area while expanding the irrigated area with the same total amount of water, thereby optimizing water productivity under the concept of deficit irrigation [57,58].

In the case of sesame crop, supplementary irrigation during the blooming and pod development stages and the drought stress mechanism of the crop to complete its lifecycle early helped to achieve better yield [59], though the yield was still less compared to the previous experiment year (Fig 10a and 10b). The crops without any water management completely failed during the year 2023. The results also highlight the importance of proper rainwater management. Storing excess rainfall and using it for supplementary irrigation during critical growth stages can help prevent crop failures and improve yields, as suggested by studies on integrated water management practices [60].

**Fig 11:**
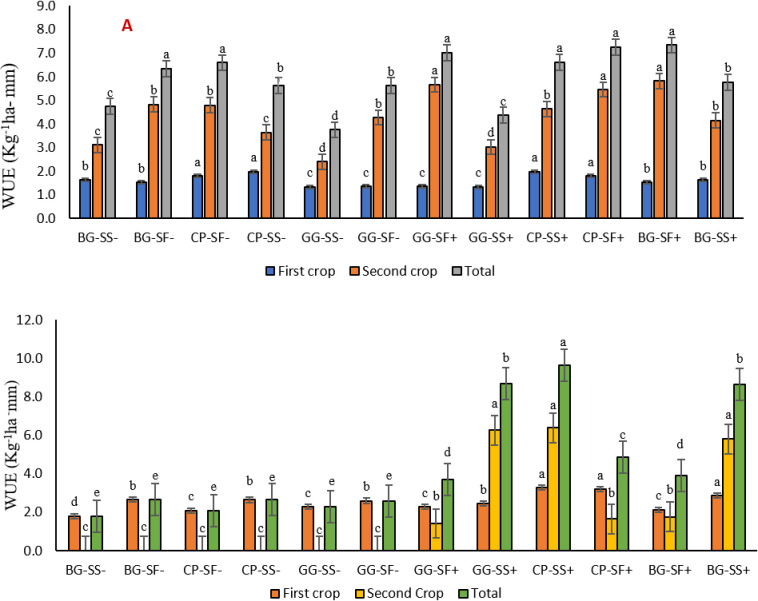
Rain water use efficiency of different crops in the system for the year 2022(A) and 2023(B).

Among the monsoon season grown crops, the highest rainwater use efficiency was obtained in the cowpea in both the years in 2022 (2.0 kg ha^-1^-mm) and 2023 (3.3 kg ha^-1^-mm). Improved initial growth, its spreading habit and increased biomass might have led to higher yields of cowpea. Cowpea is also capable of extracting stored soil moisture from deeper layers and can better withstand prolonged periods of water scarcity. [61]. The ability to put on more biomass competing with the weeds [62] might have resulted in higher water use efficiency.. Among the post monsoon crops safflower recorded highest rain water use efficiency in 2022 (5.8 kg ha^-1^-mm) and sesame in 2023 (6.4 kg ha^-1^-mm) (Fig 11a and 11b). In the second year, sesame crop completed its life cycle earlier due to dry spells and effectively utilizes the provided supplementary irrigation led to improved water use efficiency (WUE).

### 3.6. Crop productivity

**Fig 12:**
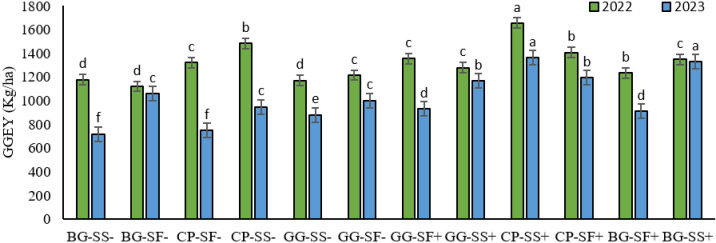
Green gram equivalent yield of cropping systems in the year 2022 and 2023.

This study assessed the performance of various double cropping systems in terms of green gram equivalent yield (GGEY). The results demonstrated that the cowpea-sesame system with rainwater management recorded the highest equivalent yield among the various double cropping systems, with a GGEY of 1655 kg ha^-1^ in 2022 and 1362 kg ha^-1^ in 2023. This system was closely followed by the cowpea-sesame system without rainwater management, which produced 1484 kg ha^-1^ in 2022. In contrast, the black gram-sesame system with rainwater management yielded 1223 kg ha^-1^ in 2023. The lowest equivalent yields were observed in the black gram-safflower system without rainwater management (1121 kg ha^-1^) in 2022, whereas black gram-sesame without rainwater management (718 kg ha^-1^) in 2023 (Fig 12). The profit gained from the systems in both the years also shows that cowpea-sesame system is sustainable over the years. Cowpea – sesame with rainwater a gross profit of ₹133334 in the first year where as ₹ 88192 in the second year (Table S1)

The better performance of sesame in 2022 and 2023 can be attributed to its drought tolerance, shorter duration of three months compared to safflower’s four months, and higher market price. The importance of rainwater management in enhancing crop yields is also evident from the results of the year 2023, as yields were achieved with rainwater management compared to systems without it that completely failed. The importance of effective rainwater management is clearly evident in this research as mentioned previously by [63].

## 4. Conclusion

The study confirms the significant advantages of diversified legume-oilseed double cropping systems in improving agricultural productivity in the semi-arid tropics, with the cowpea-sesame combination emerging as the most effective system. The cowpea-sesame system with rainwater management recorded the highest green gram equivalent yields, achieving 1655 kg ha^-1^ in 2022 and 1362 kg ha^-1^ in 2023, highlights the critical role of rainwater management in taking two crops. The better performance of sesame, due to its drought tolerance, shorter growth duration, and higher market value underscores the importance of selecting resilient crop and their varieties in the wake of climate variability. This research study also suggests that adopting effective rainwater management techniques, along with the selection of drought-tolerant crops like sesame, for sustaining agricultural productivity in these regions. Future agricultural strategies should prioritize the development of resilient cropping systems that can withstand the challenges posed by climate change, ensuring food security and economic stability for farmers in semi-arid areas globally.

## Supporting information

S1 Table Gross profit from various crops and treatments. (DOCX)
