# Supplementary material for: Diversified legume-oilseed cropping system for synergistic enhancement of yield and water use efficiency in rainfed areas of semi-arid tropics
Source: PLoS One. 2025 Feb 12;20(2):e0317373. doi: 10.1371/journal.pone.0317373 (PMC11819599; doi:10.1371/journal.pone.0317373)
Supplement: S1 Table — (DOCX) [file pone.0317373.s001.docx]

| **Treatments** | | **Profit from the I crop (2002) (₹)** | **Profit from the I crop (2003) (₹)** | **Profit from the II crop (2002) (₹)** | **Profit from the II crop (2004) (₹)** | **Total profit (2002)** | **Total profit (2003)** |
| --- | --- | --- | --- | --- | --- | --- | --- |
| BG-SS- | 67215 | | 53834 | 27544 | - | 94759 | 53834 |
| BG-SF- | 62792 | | 79493 | 27393 | - | 90186 | 79493 |
| CP-SF- | 79786 | | 56123 | 27186 | - | 106971 | 56123 |
| CP-SS- | 87753 | | 71043 | 32256 | - | 120009 | 71043 |
| GG-SS- | 67308 | | 66015 | 21288 | - | 88595 | 66015 |
| GG-SF- | 67880 | | 75158 | 24217 | - | 92097 | 75158 |
| GG-SF+ | 67880 | | 66016 | 35127 | 51 | 103007 | 66066 |
| GG-SS+ | 67308 | | 70631 | 29738 | 225 | 97045 | 70856 |
| CP-SS+ | 87753 | | 87963 | 45581 | 229 | 133334 | 88192 |
| CP-SF+ | 79786 | | 85767 | 33818 | 60 | 113604 | 85826 |
| BG-SF+ | 62792 | | 63969 | 36084 | 63 | 98876 | 64032 |
| BG-SS+ | 67215 | | 85388 | 40788 | 208 | 108003 | 85596 |

**Table S1: Gross profit from various crops and treatments**

1 **(₹)= 0.12USD**

**Cost of various crops**

| **Crops** | **Price (2022) (₹/kg)** | **Price (2023) (₹/kg)** |
| --- | --- | --- |
| Greengram | 78 | 75 |
| Black gram | 66 | 69 |
| Cowpea | 59 | 62 |
| Sesame | 78 | 96 |
| Safflower | 53 | 59 |
